# Role of Resveratrol and Selenium on Oxidative Stress and Expression of Antioxidant and Anti-Aging Genes in Immortalized Lymphocytes from Alzheimer’s Disease Patients

**DOI:** 10.3390/nu11081764

**Published:** 2019-07-31

**Authors:** Marta Cosín-Tomàs, Júlia Senserrich, Marta Arumí-Planas, Carolina Alquézar, Mercè Pallàs, Ángeles Martín-Requero, Cristina Suñol, Perla Kaliman, Coral Sanfeliu

**Affiliations:** 1 Institut d’Investigacions Biomèdiques de Barcelona (IIBB), Consejo Superior de Investigaciones Científicas (CSIC), 08036 Barcelona, Spain; 2 Department of Human Genetics, Research Institute of the McGill University Health Centre, Montreal, QC H3A 0C7, Canada; 3 Department of Molecular Biomedicine, Centro de Investigaciones Biológicas, CSIC, 28040 Madrid, Spain; 4 Faculty of Pharmacy and Food Sciences, Institut de Neurociències, Universitat de Barcelona, 08028 Barcelona, Spain; 5 CIBER de Enfermedades Neurodegenerativas (CIBERNED), Instituto de Salud Carlos III, 28031 Madrid, Spain; 6 CIBER de Epidemiología y Salud Pública (CIBERESP), Instituto de Salud Carlos III, 28031 Madrid, Spain; 7 Institut d’Investigació Biomèdica August Pi i Sunyer (IDIBAPS), 08036 Barcelona, Spain; 8 Faculty of Health Sciences, Universitat Oberta de Catalunya, 08018 Barcelona, Spain

**Keywords:** Alzheimer’s disease, immortalized B lymphoblastoid cell lines, resveratrol, selenium, oxidative stress, aging, gene expression

## Abstract

Oxidative damage is involved in the pathophysiology of age-related ailments, including Alzheimer’s disease (AD). Studies have shown that the brain tissue and also lymphocytes from AD patients present increased oxidative stress compared to healthy controls (HCs). Here, we use lymphoblastoid cell lines (LCLs) from AD patients and HCs to investigate the role of resveratrol (RV) and selenium (Se) in the reduction of reactive oxygen species (ROS) generated after an oxidative injury. We also studied whether these compounds elicited expression changes in genes involved in the antioxidant cell response and other aging-related mechanisms. AD LCLs showed higher ROS levels than those from HCs in response to H_2_O_2_ and FeSO_4_ oxidative insults. RV triggered a protective response against ROS under control and oxidizing conditions, whereas Se exerted antioxidant effects only in AD LCLs under oxidizing conditions. RV increased the expression of genes encoding known antioxidants (catalase, copper chaperone for superoxide dismutase 1, glutathione S-transferase zeta 1) and anti-aging factors (sirtuin 1 and sirtuin 3) in both AD and HC LCLs. Our findings support RV as a candidate for inducing resilience and protection against AD, and reinforce the value of LCLs as a feasible peripheral cell model for understanding the protective mechanisms of nutraceuticals against oxidative stress in aging and AD.

## 1. Introduction

Alzheimer’s disease (AD) is a neurodegenerative disease which is the main cause of dementia worldwide, and it is currently incurable. It is characterized by a loss of memory and progressive cognitive, functional, and behavioral decline that interferes with daily life [1]. It is now well recognized that the pathogenesis begins up to one or two decades before the onset of the clinical symptoms [2]. Therefore, understanding the mechanisms that lead to the progression of the disease is essential to establish an early diagnosis and slow or prevent its progression.

The two hallmarks of AD are the accumulation of extracellular senile plaques formed by amyloid beta (Aβ) peptides, and the accumulation of hyperphosphorylated tau aggregates that form neurofibrillary tangles (NFTs) inside neurons [3]. In addition to plaques and NFTs, oxidative damage to proteins, nucleic acids, and lipids plays a key role in the pathophysiology of the disease, as in most age-related ailments [4]. It has been reported that Aβ promotes the generation of reactive oxygen species (ROS), either directly or indirectly, by triggering N-methyl-D-aspartate receptor-dependent Ca^2+^ influxes and leading to mitochondrial dysfunction. However, it has been postulated that Aβ accumulation may be a consequence of oxidative stress and that Aβ and tau act as antioxidants in AD (reviewed by Sutherland et al. [5]). Although this issue is still unclear, a wide range of studies have shown that the imbalance between the production of ROS, on the one hand, and antioxidant defenses, on the other, contribute considerably to the pathogenesis and progression of AD [4,6,7,8]. In fact, considerable attention in AD research has been focused on identifying compounds capable of scavenging excess ROS.

In particular, resveratrol (RV; trans-3,4′,5-trihydroxystilbene) and selenium (Se), which are both nutraceuticals with antioxidant properties that can permeate the brain blood barrier, seem to have therapeutic potential as neuroprotective agents [9,10]. RV is a polyphenol that is mainly found in some fruits such as blueberries, blackberries and grapes, and also in peanuts. It has been shown that RV mimics the anti-aging and neuroprotective effects of caloric restriction through sirtuin 1 (SIRT1) mechanisms [11]. RV indirectly activates SIRT1 through cAMP signaling that leads to activation of the 5′ AMP-activated protein kinase (AMPK)/SIRT1 pathway [12,13]. Furthermore, both in vitro and in vivo experimental AD studies have suggested that RV activates the SIRT1 pathway as its main neuroprotective mechanism [14,15,16,17]. However, RV may partially act through other mechanisms, as demonstrated by in vitro treatments in the presence of the SIRT1 inhibitor sirtinol, where RV neuroprotection was only partially abolished [18]. In this regard, RV has potent antioxidant properties through direct scavenging of ROS. Some clinical trials have shown that resveratrol is safe, well-tolerated, and is capable of decreasing neuroinflammation and modifying some AD biomarkers, such as cerebrospinal fluid Aβ40 and Aβ42 [19,20].

Meanwhile, Se is an essential micronutrient for brain function that plays a critical role in multiple metabolic pathways, including those involved in antioxidant defense in organisms [21]. Se is a component of antioxidant enzymes, such as glutathione peroxidase, and there are a number of other selenoenzymes and selenoproteins. There are two different commonly occurring forms of Se in nature, selenite (Se (IV)) and selenate (Se (VI)), and both have been studied in the context of the prevention of AD onset and progression. Studies have shown that diets supplemented with these components can play a neuroprotective role in AD experimental models [22,23,24]. For example, Se (IV) can reduce the amount of Aβ plaques [23] and Se (VI) may reduce hyperphosphorylation of tau [24]. Studies in humans have found a significant decrease of Se in AD brains or blood cells, compared to controls [25,26]. Therefore, both RV and Se diet supplementation are promising strategies to combat aging and AD.

Given that several peripheral and systemic abnormalities interact with the brain and influence the development and progression of the pathology, it has been suggested that AD may be considered a systemic disease [27]. In fact, many authors have shown that not only do components of the nervous system from AD patients present increased oxidative stress markers compared to healthy controls (HCs), but so too do lymphocytes [28]. This has prompted some authors to use lymphoblastoid cell lines (LCLs) from AD patients and HCs as a suitable and more feasible model to study the disease in vitro. These human cell lines arise from peripheral B lymphocytes infected in vitro with the Epstein–Barr virus; a process that immortalizes them [29]. Some studies have already shown alterations in the cell cycle, proliferative activity and Aβ processing, as well as higher oxidative stress in AD than control-derived LCLs [30,31,32,33,34,35,36,37,38,39].

Within this context, we use LCLs from AD patients and HCs to investigate the potential role of RV and both Se (IV) and Se (VI) in the reduction of ROS generated after an oxidative injury. We also examine whether these compounds elicit expression changes in genes involved in the antioxidant cell response and other aging-related mechanisms. We found that AD LCLs showed a lower capacity of response against oxidative injuries than HC LCLs, as expected. Furthermore, RV triggered a protective response against ROS under control and oxidizing conditions and increased the expression of gene coding for known antioxidants and anti-aging factors; whereas Se exerted antioxidant effects only in AD LCLs under oxidizing conditions. Our findings support RV as a powerful compound with preventive and therapeutic properties against redox and aging alterations of AD and reinforce the value of LCLs as a human cell model for studying the protective mechanisms of nutraceuticals.

## 2. Materials and Methods

### 2.1. Cell Lines

Immortalized lymphocytes from AD patients from the Department of Neurology of the University Hospital Doce de Octubre (Madrid, Spain) and age-matched HCs, were used for this study. AD patients were at a moderate stage of the disease and presented values between 10 and 18 in the Mini-Mental State Examination. Details of the informed consent and technical procedures for the establishment of LCLs from peripheral blood samples were previously reported [32]. The cells were cultured in Roswell Park Memorial Institute (RPMI)-1640 (Biowest, Nuaillé, France, #L0500), which contained 2 mM L-glutamine, supplemented with 10% fetal bovine serum (FBS; Gibco, Pailey, Scotland, #10270) and 1% penicillin/streptomycin (Gibco, #15070) or 0.1% gentamicin (Gibco, #15750-045). LCLs were grown in suspension inside T25 flasks in an upright position, in 8 mL of completed medium per flask and at a density of 1 × 10^6^ cells/mL. They were maintained in a humidified 5% carbon dioxide incubator at 37 °C. The culture medium was routinely changed every 2 days by removing 4 mL of the medium from above the cells and replacing it with an equal volume of fresh medium. The cell lines were routinely tested for the absence of mycoplasma contamination (Mycoplasma Gel Detection Kit; Biotools, Madrid, Spain, #4542). For the experiments, cells were seeded in 2 mL tubes with 1 mL of medium without FBS for the time required before each experiment at a concentration of 3 × 10^5^ cells/mL. All the cell culture plastic was from Nunc™ (ThermoFisher Scientific, Waltham, MA, USA).

### 2.2. Characterization of Oxidative Stress by DCFH-DA (2′7′-dichloro-dihydro-fluorescein diacetate) Assay

The most widely used probe for the detection of oxidative species in living cells is 2′7′-dichloro-dihydro-fluorescein diacetate (DCFH-DA). It is a non-fluorescent cell-permeable molecule. Within cells, the acetate groups are hydrolyzed by intracellular esterases, leading to 2′,7′-dichloro-dihydro-fluorescein (DCFH). The presence of ROS, mainly hydroperoxides, oxidizes DCFH to dichlorofluorescein (DCF), which is highly fluorescent [40]. To use this technique, cells from 2–3 different AD or HC LCLs were seeded at a cell density of 3 × 10^5^ cells/mL in T25 flasks, the day before the experiment, in FBS-free medium. For the test, the cells were gently homogenized with the medium and 1 mL was transferred to different tubes. After centrifugation at 1000 rpm for 5 min, the cells were resuspended with 400 µL of 4-(2-hydroxyethyl)-1-piperazineethanesulfonic acid (HEPES)-buffered saline solution (HBSS) and 4 µL of 100× DCFH-DA (10 µM final concentration; Molecular Probes, Leiden, The Netherlands, #D-399) or 4 µL of HBSS for the negative controls. Negative controls were used to obtain the background fluorescence data and were processed in parallel throughout the experiment. After suspension, all the samples were incubated for 20 min with gentle shaking at 37 °C in the dark to allow the DCFH-DA to load into the cells. After centrifugation for 5 min at 200 rcf, the supernatant was removed, and the cells were resuspended in 400 µL of HBSS containing the corresponding treatments (performed in triplicates). The different conditions were: control treatment with HBSS only, and different concentrations of the oxidizing agents H_2_O_2_ (200 µM, 500 µM, and 1000 µM; Sigma, St Louis, MO, USA, #216763) and FeSO_4_ (1 µM, 5 µM, and 25 µM; Sigma, #F7002) in HBSS. Then, all the samples were incubated for 1 h with gentle shaking at 37 °C in the dark. Next, 44 µL of 10× lysis buffer was added to each tube. All the samples were homogenized and centrifuged at 10,000 rcf for 10 min, and 200 µL of the supernatant was transferred to a 96-well plate, making two replicates per sample. Cell fluorescence was determined using a SPECTRAmax GEMINI XS microplate reader fluorimeter (Molecular Devices, San Jose, CA, USA), with a wavelength of excitation and emission of 485 nm and 530 nm, respectively. Finally, in order to control for the cellular protein content, 50 µL of 2 N NaOH solution was added to the cell pellet for future processing using the Bradford protein assay (Bio-Rad Protein Assay Dye Reagent Concentrate; Bio-Rad, Hercules, CA, USA, #500-0006).

### 2.3. DCFH-DA Assay to Study Se (IV), Se (VI,) and RV Antioxidant Effects

The same protocol as for the DCFH-DA assay described above was applied with some changes. Cells from 2–3 different AD or HC LCLs were seeded at a density of 3 × 10^5^ cells/mL in 2 mL tubes. Twenty µL of each protective treatment was added to the corresponding tubes (performed in triplicate). The concentrations of the protective compounds were obtained from the literature and tested in preliminary studies not to affect cell growth or viability. The different protective treatments were: Se (IV) (Sigma, #S5261) at 5 and 10 µM, Se (VI) (Sigma, #S0882) at 100 and 200 µM, and RV (Sigma, #R5010) at 10 and 50 µM. RV, Se (IV), and Se (VI) were solubilized with DMSO (0.1%) or HBSS, respectively. Afterwards, DMSO was added to all the experimental conditions (0.1%). After overnight incubation (18 h), the samples were centrifuged at 1000 rpm for 5 min; cells were resuspended with 400 µL HBSS and 4µL of 100× DCFH-DA. Eight µL of each protective treatment was again added to maintain the corresponding concentrations. Then, the samples were incubated for 20 min with gentle shaking at 37 °C in the dark. After centrifugation for 5 min at 200 rcf, the supernatant was removed, and the cells were resuspended in 400 µL of the corresponding protective treatment and/or the agents used to induce oxidative stress: H_2_O_2_ and FeSO_4_. The final experimental conditions were: control, 1000 µM H_2_O_2_ or 5 µM FeSO_4_, with 8 µL of Se (IV), Se (VI), or RV. The samples were incubated for 1 h with gentle shaking at 37 °C in the dark. Next, 44 µL of lysis buffer 10× was added to each tube. All the points were homogenized and after centrifugation at 10,000 rcf for 10 min, 200 µL of the supernatant was transferred to a 96-well plate, making 2 replicates for each sample. Finally, in order to control the cellular protein content, 50 µL of 2 N NaOH solution was added to the cell pellet for future processing using the Bradford protein assay.

### 2.4. Gene Expression Analysis

Cells from 2–3 different AD or HC LCLs were transferred to 2 mL tubes containing FBS-free medium and incubated for 18 h at a concentration of 3 × 10^5^ cells/mL with the corresponding treatment (four replicates per experimental condition). The samples were removed from the incubator and rapidly centrifuged at 1300 rpm for 5 min and washed with 200 µL of PBS. Then, the samples were centrifuged again at the same speed for 5 min, the supernatant was removed, and cells were resuspended with 20 µL of PBS followed by 180 µL of RNA later (Sigma, #R0901). The samples were stored at 4 °C. The different experimental conditions consisted of: non-treatment control, Se (IV) at 10 µM, Se (VI) at 200 µM, and RV at 50 µM. RV, Se (IV), and Se (VI) were solubilized with DMSO (0.1%) and HBSS, respectively. DMSO was added in all experimental conditions. The analysis of gene expression was performed in the absence of oxidative challenges.

### 2.5. mRNA Purification

The four samples corresponding to the same conditions were pooled in one single tube. Then, RNA later was removed, and RNA was extracted using mirVana™ miRNA Isolation Kits (Life Technologies, Carlsbad, CA, USA, #AM1561), following the manufacturer’s instructions to obtain total RNA, including small RNA. The quantity and quality of the RNA samples was determined using a ND-1000 spectrophotometer (NanoDrop Technologies, Wilmington, DE, USA). Samples with low concentrations of RNA were concentrated using the SpeedVac vacuum system (Savant, ThermoFisher, Waltham, MA, USA). Samples were stored at −80 °C until further use.

### 2.6. cDNA Reverse Transcription

Random-primed cDNA synthesis was performed at 37 °C starting with 0.3 μg of RNA, using high-capacity cDNA Reverse Transcription Kits (Life Technologies, #4368814). All the samples were diluted at a 1:4 ratio and stored at −20 °C until further use.

### 2.7. Real-Time Quantitative PCR

Gene expression of candidate genes was determined using TaqMan Fluorescein amidite (FAM)-labeled specific probes (Applied Biosystems, Foster City, CA, USA) and Quantimix Easy Probe kits (Biotools, #10.601-4149) in an RFX96TM Real-time system (Bio-Rad). Samples were analyzed in duplicate. Results were normalized to phosphoglycerate kinase 1 (*PGK1*) and beta-2-microglobulin (*B2M*) gene expression levels. A list of probes is provided in Supplementary Table S1.

### 2.8. Statistical Analysis

Statistical analysis was performed using GraphPad Prism 5.01 software (GraphPad Software, La Jolla, CA, USA). Analysis was via two-way ANOVA. Significance values were given for the two factors: treatment (Tr) and disease (Ds), and for the interaction Tr × Ds. A post hoc Tukey’s test, or Fisher’s Least Significant Difference (LSD) test, was applied after a significant Tr effect (since this factor has more than two levels) and after a significant interaction. All the values are shown as mean ± standard error (SEM). Statistical outliers (≥ two standard deviations from the mean) were removed from the analysis. *P*-values ≤ 0.05 were considered statistically significant.

## 3. Results

### 3.1. Characterization of HC and AD LCLs in Response to Acute Exposure of Oxidizing Agents

**Figure 1:**
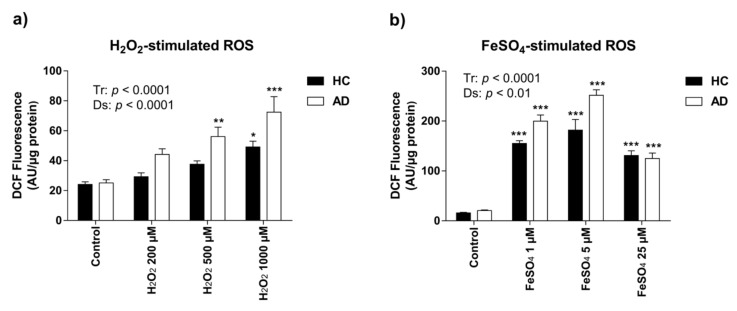
Reactive oxygen species (ROS) generation by lymphoblast cell lines in response to acute exposure (1 h) to oxidizing agents measured by 2′7′-dichloro-dihydro-fluorescein diacetate (DCFH-DA) assay. (**a**) H_2_O_2_–stimulated ROS. One h of treatment with H_2_O_2_ induced oxidative stress, showing significant greater ROS generation in Alzheimer’s disease (AD) than in healthy control (HC) lymphoblasts. Two-way ANOVA indicated an effect of both treatment (Tr) (F(3,16) = 19.52; *p* < 0.0001) and disease (Ds) (F(1,16) = 17.21; *p* < 0.001); (**b**) FeSO_4_-stimulated ROS. FeSO_4_ induced oxidative stress. Two-way ANOVA indicated effects of both Tr (F(3,16) = 122.2 and *p* < 0.0001) and Ds (F(1,16) = 13.23; *p* < 0.01); and an interaction Tr × Ds (F(3,16) = 5.121; *p* < 0.05). Values are mean ± SEM of three independent experiments with *n* = 3–5/experiments on two different cell lines per group. *P*-values of Tukey’s post hoc tests for each experimental condition relative to the control treatment within each group (AD or HC) are presented as: * *p* < 0.05, ** *p* < 0.01, and *** *p* < 0.001.

Intracellular ROS formation in HC and AD LCLs after 1 h exposure to different concentrations of the oxidizing agents H_2_O_2_ (200 µM, 500 µM, and 1000 µM) and FeSO_4_ (1 µM, 5 µM, and 25 µM) was measured by the DCFH-DA assay. Higher concentrations of H_2_O_2_ produced more oxidative stress than lower doses (Tr, F(3,16) = 19.52, *p* < 0.0001) (Figure 1a). Notably, there was a significantly higher ROS production in AD than in HC lymphoblasts (Ds, F(1,16) = 17.21, *p* < 0.0001) (Figure 1a). Treatment with H_2_O_2_ became significant at 500 µM and 1000 µM H_2_O_2_ in AD and HC lymphoblasts, respectively, indicating that AD lymphoblasts are more sensitive to oxidative stress insults (Tukey’s post hoc tests, control vs. 500 µM H_2_O_2_: HC *p* = ns, AD *p* < 0.01; control vs. 1000 µM H_2_O_2_: HC *p* < 0.01, AD *p* < 0.0001).

Exposure to higher concentrations of FeSO_4_ triggered an increase in the production of ROS in both groups (Tr, F(3,16) = 122.2, *p* < 0.0001) (Figure 1b). However, it is important to note that with the highest concentration of FeSO_4_ used (25 µM), ROS levels did not further increase but were rather lower than with the 1 µM and 5 µM doses, which suggests that this concentration is too high for increasing the metabolic response to a higher ROS production in these particular cell lines. As with H_2_O_2_, there was a significantly higher ROS production in AD than in HC lymphoblasts (Ds, F(1,16) = 13.23, *p* < 0.01) (Figure 1b).

Therefore, AD LCLs showed higher ROS generation than HC LCLs after both peroxide-induced and iron-catalyzed oxidative injuries.

### 3.2. Protective Effect of RV and Se against ROS Production in HC and AD LCLs

In order to explore the protective action of RV, Se (IV), and Se (VI) treatments on oxidative stress, DCFH-DA assays were conducted in both HC and AD LCLs. Two different concentrations of each compound were used as follows: 5 µM and 10 µM Se (IV), 100 µM and 200 µM Se (VI), and 10 µM and 50 µM RV. All the concentrations were into the range of those used in a number of previously reported in vitro studies. Specifically, we selected the highest effective concentrations that did not inhibit viability and cell growth in human lymphocytes [41,42,43,44]. Compounds were incubated overnight (18 h) and immediately tested for their effects on inhibiting ROS generation for a further 1 h under control (vehicle) and oxidizing conditions (1000 µM H_2_O_2_ and 5 µM FeSO_4_).

**Figure 2:**
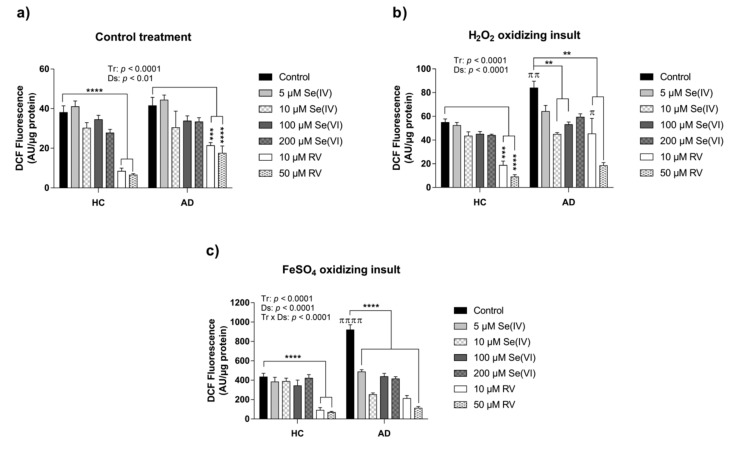
Intracellular ROS after 18 h of exposure to selenite (Se (IV)), selenate (Se (VI)), and resveratrol (RV) measured by DCFH-DA assay in HC and AD lymphoblasts cell lines. (**a**) DCFH-DA assay with Se (IV), Se (VI), and RV treatments in combination with the vehicle. Two-way ANOVA indicated overall treatment (Tr) and sisease (Ds) effects: (F(6,57) = 26.95; *p* < 0.0001) and (F(1,57) = 9.056; *p* < 0.01), respectively; (**b**) DCFH-DA assay with Se (IV), Se (VI), and RV treatments in combination with H_2_O_2_. Two-way ANOVA indicated overall Tr and Ds effects: (F(6,57) = 21.07; *p* < 0.0001) and (F(1,57) = 23.68; *p* < 0.0001), respectively; (**c**) DCFH-DA assay with Se (IV), Se (VI), and RV treatments in combination with FeSO_4_. Two-way ANOVA indicated overall Tr and Ds effects, as well as an interaction Tr × Ds: (F(6,57) = 48.69; *p* < 0.0001), (F(1,57) = 22.86; *p* < 0.0001), and (F(6,57) = 11.74; *p* < 0.0001), respectively. Values are mean ± SEM of six independent experiments with n = 2/experiment on two different cell lines per group. HC and AD stand for healthy control and Alzheimer’s disease, respectively. *P*-values of Tukey’s post hoc tests are presented as: ** *p* < 0.01, *** *p* < 0.001, and **** *p* < 0.001 for comparisons between treatments within each group (HC or AD); and as: ^π^ *p* < 0.05, ^ππ^ *p* < 0.01, and ^ππππ^ *p* < 0.001, for comparisons between groups within each experimental condition.

Two-way ANOVA analysis indicated a Tr and Ds factor effect when the DCFH-DA assay was conducted with Se (IV), Se (VI), and RV in control conditions (Tr, F(6,57) = 26.95; *p* < 0.0001; Ds, F(1,57) = 9.056; *p* < 0.01) (Figure 2a). Particularly, the decrease in ROS was significantly triggered by RV according to Tukey’s post hoc tests (HC: control vs. 10 or 50 µM RV, *p* < 0.0001; AD: control vs. 10 µM RV, *p* < 0.001, control vs. 50 µM RV, *p* < 0.0001) (Figure 2a).

Similarly, under oxidizing conditions triggered by both H_2_O_2_ and FeSO_4_, there was a general Tr and Ds effect, according to two-way ANOVA analysis for H_2_O_2_ (Tr, F(6,57) = 21.07; *p* < 0.0001; Ds, F(1,57) = 23.68; *p* < 0.0001) and FeSO_4_ (Tr, F(6,57) = 48.69; *p* < 0.0001; Ds, F(1,57) = 22.86; *p* < 0.0001) (Figure 2b,c).

Interestingly, in the case of H_2_O_2_ cell culture experiment, RV was the only treatment that lowered ROS production in the HC LCLs, whereas Se (IV), Se (VI), and RV treatments were protective for AD LCLs (HC: control vs. 10 µM RV, *p* < 0.001, control vs. 50 µM RV, *p* < 0.0001; AD: control vs. 10 µM Se (IV), 100 µM Se (VI), 10 µM RV, and 50 µM RV, all *p* < 0.01) (Figure 2b). Regarding differences between HC and AD groups at the same experimental condition, Tukey’s post hoc tests indicated higher ROS levels in AD LCLs compared to HC LCLs under H_2_O_2_ alone and under H_2_O_2_ plus 10 µM RV treatment (control: HC vs. AD, *p* < 0.01; 10µM RV treatment: HC vs. AD, *p* < 0.05) (Figure 2b).

Notably, in the case of lymphoblasts exposed to FeSO_4_, there was a significant interaction Tr × Ds (F(6,57) = 11.74; *p* < 0.0001) (Figure 2c). As in the case of H_2_O_2_, RV triggered a reduction of ROS in the HC LCLs (control vs. 10 or 50 µM RV, *p* < 0.0001), whereas all treatments significantly decreased ROS production in AD LCLs (AD: control vs. 5 or 10 µM Se (IV), 100 or 200 µM Se (VI), 10 or 50 µM RV, *p* < 0.0001) (Figure 2c).

Overall, RV triggered a general protective response against ROS under both control and oxidizing conditions whereas Se exerted antioxidant effects only in AD cell lines under oxidizing conditions. AD LCLs showed higher ROS levels than HC LCLs in the presence of oxidative agents, which is consistent with results from the first experiments described above. Although we observed a consistent protective effect of RV and Se against oxidative stress, additional characterization would be required to confirm the absence of harmful effects of these compounds on other cellular pathways when administered in combination with oxidative agents (i.e., H_2_O_2_ or FeSO_4_).

### 3.3. Transcriptional Changes in Oxidative Stress-Related Genes Induced by Se (IV), Se (VI), and RV in HC and AD LCLs

Lower levels of antioxidant enzymes and other first-line endogenous defenses against oxidative stress have been reported in AD [4,6]. Furthermore, according to several studies of animals and other in vitro models, RV and Se can induce an antioxidant response by triggering transcriptional changes in genes involved in the cellular antioxidant system [45,46]. In order to study these particular mechanisms in our model, we incubated HC and AD LCLs for 18 h with either the vehicle alone or the highest concentration previously tested against oxidative injury, namely 10 µM Se (IV), 200 µM Se (VI), and 50 µM RV. We then measured the mRNA levels of a battery of genes known to be involved in oxidative stress processes (Table 1, Supplementary Table S1).

**Figure 3:**
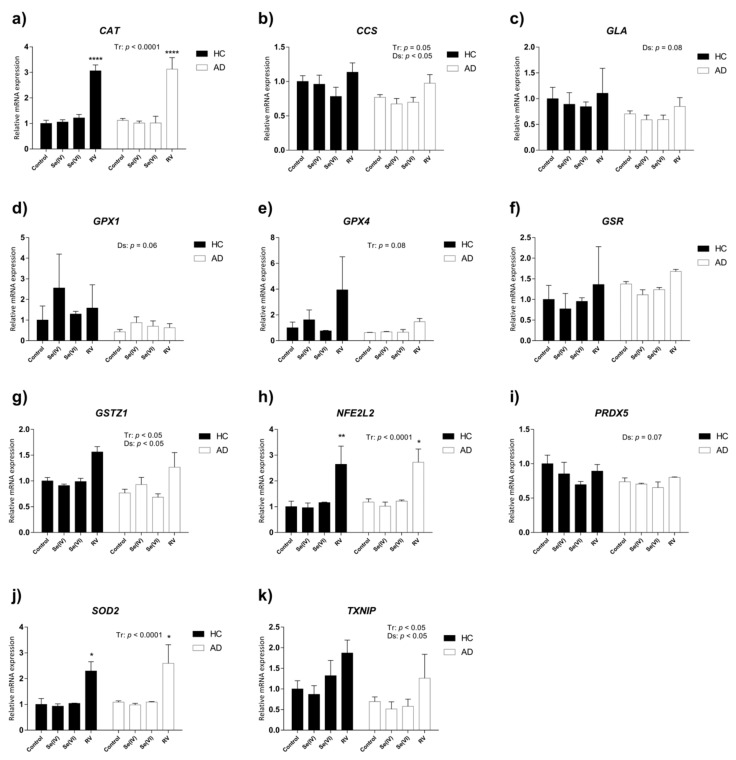
Relative expression of genes involved in oxidative stress in AD and HC lymphoblast cell lines treated with selenite (Se (IV)), selenate (Se (VI)), and resveratrol (RV). Gene expression analysis by real-time PCR from lymphoblast mRNA using TaqMan Fluorescein amidite (FAM)-labeled specific probes and normalized with the mean of both housekeeping genes: phosphoglycerate kinase 1 (*PGK1*) and beta-2-microglobulin (*B2M*). (**a**) Catalase (*CAT*); (**b**) copper chaperone for *SOD1* (*CCS*); (**c**) alpha galactosidase (*GLA*); (**d**) glutathione peroxidase 1 (*GPX1*); (**e**) glutathione peroxidase 4 (*GPX4*); (**f**) glutathione reductase (*GSR*); (**g**) glutathione S-transferase zeta 1 (*GSTZ1*); (**h**) nuclear factor (erythroid-derived 2)-like 2 (*NEF2L2*); (**i**) peroxiredoxin 5 (*PRDX5*); (**j**) superoxide dismutase 2 (*SOD2*); (**k**) thioredoxin interacting protein (*TXNIP*). *P*-values for two-way ANOVA analysis are indicated at the top or right area of the graph. Tr: treatment effect; Ds: disease effect. *P*-values of Tukey’s post hoc tests for each group (relative to control treatment) are indicated in the graphs as: + *p* < 0.1, * *p* < 0.05, ** *p* < 0.01, **** *p* < 0.0001. Values are mean ± SEM of two to four independent experiments with n = 2/experiment on two different cell lines per group. HC and AD stand for healthy control and Alzheimer’s disease, respectively.

Two-way ANOVA revealed that catalase *(CAT)*, glutathione S-transferase zeta 1 *(GSTZ1)*, nuclear factor (erythroid-derived 2)-like 2 *(NFE2L2)*, superoxide dismutase 2 *(SOD2)*, and thioredoxin interacting protein *(TXNIP)* were upregulated due to the treatments (Table 1, Figure 3a,g–j). Particularly, RV significantly increased *CAT*, *NFE2L2*, and *SOD2* in both HC and AD LCLs, whereas the change in expression of *GSTZ1* and *TXNIP* did not reach significance according to Tukey’s post hoc tests (*CAT*: HC or AD control vs. HC or AD RV *p* < 0.0001; *NFE2L2*: HC or AD control vs. HC or AD RV *p* < 0.01; *SOD2*: HC control vs. HC RV *p* < 0.05, AD control vs. AD RV *p* < 0.01). In the case of copper chaperone for SOD1 *(CCS)* and glutathione peroxidase 4 *(GPX4)*, there was a tendency towards upregulation by treatment according to two-way ANOVA (Table 1, Figure 3b,e).

Meanwhile, we observed a global decrease in gene expression of *CCS*, *GSTZ1*, and *TXNIP* in AD lymphoblasts compared to HC LCLs, according to two-way ANOVA analysis (Table 1, Figure 3b,g,k). No interactions between treatment and disease were detected for any of the genes studied.

As a whole, the treatment with RV demonstrated the capacity to induce several antioxidant and ROS detoxifying genes in HC and AD LCLs, whereas the effects of Se were minor and not statistically significant. Furthermore, AD LCL antioxidant defenses seemed impaired according to the lower expression of some of the genes in comparison to HC LCLs.

### 3.4. Transcriptional Changes in Age-Related Genes Induced by Se (IV), Se (VI), and RV in HC and AD LCLs

Se and RV are reported to have a beneficial effect on lifespan, telomere length, DNA damage, inflammation, senile plaque formation, hyperphosphorylation of tau, and other processes associated with aging and AD. Although the exact mechanisms of action remain unclear, some authors have observed changes in aging-related genes as a consequence of RV and Se treatments in different models [45,46,58]. To further explore the mechanisms by which these compounds exert potential therapeutic effects in AD, we treated HC and AD lymphoblasts with either the vehicle alone or the previously tested concentrations of 10 µM Se (IV), 200 µM Se (VI), and 50 µM RV. We then measured the mRNA levels of a battery of genes involved in age-related processes or AD pathology (Table 2, Supplementary Table S1).

**Figure 4:**
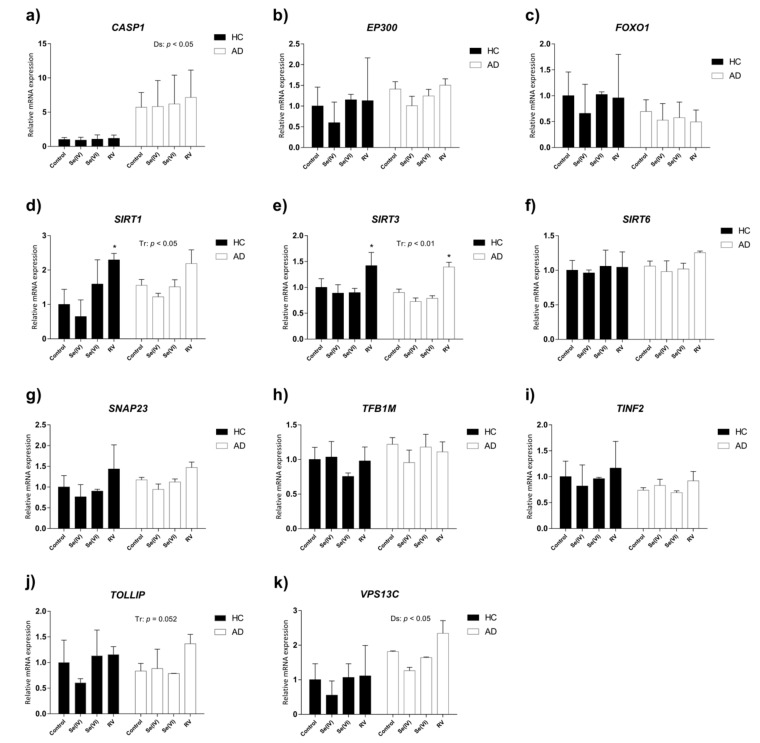
Relative expression of age-related genes in AD and HC lymphoblast cell lines treated with selenite (Se (IV)), selenate (Se (VI)), and resveratrol (RV). Gene expression analysis by real-time PCR from lymphoblast mRNA using TaqMan FAM-labeled specific probes and normalized with the mean of both housekeeping genes: phosphoglycerate kinase 1 (*PGK1*) and beta-2-microglobulin (*B2M*). (**a**) Caspase 1 (*CASP1*); (**b**) E1A binding protein p300 (*EP300*); (**c**) forkhead box O1 (*FOXO1*); (**d**) sirtuin 1 (*SIRT1*); (**e**) sirtuin 3 (*SIRT3*); (**f**) sirtuin 6 (*SIRT6*); (**g**) synaptosome associated protein 23 (*SNAP23*); (**h**) transcription factor B1, mitochondrial (*TFB1M*); (**i**) TERF1-interacting nuclear factor 2 (*TINF2*); (**j**) vacuolar protein sorting 13 homolog C (*VPS13A*). *P*-values for two-way ANOVA are indicated at the top or on the right. Tr: treatment effect; Ds: disease effect. *P*-values of Fischer’s LSD post hoc test for each group (relative to control treatment) are indicated as: * *p* < 0.05. Values are mean ± SEM of two to four independent experiments with *n* = 2/experiment on two different cell lines per group. HC and AD stand for healthy control and Alzheimer’s disease, respectively.

Two-way ANOVA analysis revealed that *SIRT1* and *SIRT3* were upregulated by treatment (Table 2, Figure 4e). Post hoc analysis, however, only yielded significance for RV treatment using Fisher’s LSD test, which does not correct for multiple comparisons (*SIRT1*: HC control vs. HC RV, *p* < 0.05; *SIRT3*: HC or AD control vs. HC or AD RV, *p* < 0.05). Notably, both caspase 1 (*CASP1*) and vacuolar protein sorting 13 homolog C *(VPS13C)* were increased in AD compared to HC lymphoblasts according to two-way ANOVA analysis (Table 2, Figure 4a,k). No interactions between treatment and disease were detected for any of the genes studied.

Here we found upregulation of the pro-survival and neuroprotection sirtuin genes *SIRT1* and *SIRT3* by RV in both LCLs. However, the higher levels of the pro-apoptotic *CASP1* gene and proteostasis-related *VPS13C* gene in AD LCLs were not reversed by either RV or Se treatment.

### 3.5. Characterization of Transcriptional Changes Induced by RV at Different Concentrations on Selected Candidate Genes in HC and AD LCLs

RV showed higher potency in upregulating gene expression of key protective genes than Se compounds in our experimental conditions. Therefore, to better explore the protective mechanisms of RV against aging and AD in this human LCL model, we performed a concentration–response study of RV. To this end, AD and HC lymphoblasts were treated for 18 h with the vehicle alone or RV at a concentration of 10 µM, 20 µM, and 50 µM. We measured mRNA levels of those genes that previously showed a significant change for Tr or Ds effect (Figure 3 and Figure 4).

**Figure 5:**
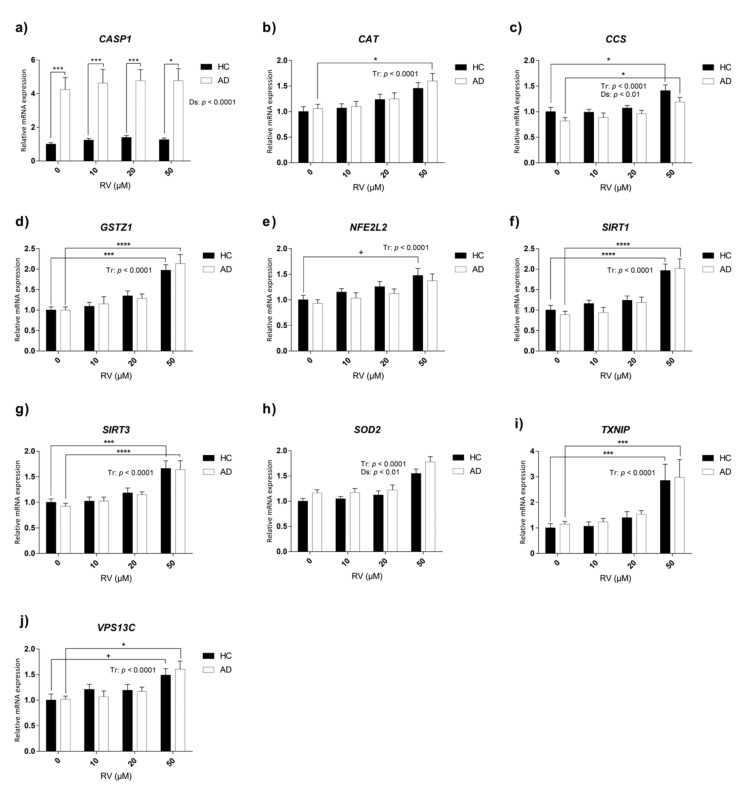
Relative expression of candidate genes involved in oxidative stress and aging in AD and HC lymphoblast cell lines treated with different concentrations of resveratrol (RV). Gene expression analysis by real-time PCR from lymphoblast mRNA using TaqMan FAM-labeled specific probes and normalized with the mean of both housekeeping genes: phosphoglycerate kinase 1 (*PGK1*) and beta-2-microglobulin (*B2M*). (**a**) Caspase 1 (*CASP1*); (**b**) catalase (*CAT*); (**c**) copper chaperone for SOD1 (*CCS*); (**d**) glutathione S-transferase zeta 1 (*GSTZ1*); (**e**) nuclear factor (erythroid-derived 2)-like 2 (*NFE2L2*); (**f**) sirtuin 1 (*SIRT1*); (**g**) sirtuin 3 (*SIRT3*); (**h**) superoxide dismutase 2 (*SOD2*); (**i**) thioredoxin-interacting protein (*TXNIP*); (**j**) vacuolar protein sorting 13 homolog C (*VPS13C*). *P*-values for two-way ANOVA analysis are indicated at the top or on the right. Tr: treatment effect; Ds: disease effect. *P*-values for Tukey’s post hoc tests are indicated as: + *p* < 0.1, * *p* < 0.05, *** *p* < 0.001, **** *p* < 0.0001. Values are mean ± SEM of seven to thirteen independent experiments with *n* = 2/experiment on two different cell lines per group. HC and AD stand for healthy control and Alzheimer’s disease, respectively.

Two-way ANOVA analysis indicated a general upregulation of *CAT*, *CCS*, *GSTZ1*, *NFE2L2*, *SIRT1*, *SIRT3*, *SOD2*, *TXNIP*, and *VPS13C* in both AD and HC LCLs due to the treatment with RV (Table 3, Figure 5b–j). However, according to Tukey’s post hoc tests, it was the highest concentration of RV that promoted significant increases in the expression of these genes (*CCS*: HC 0 µM vs. HC 50 µM RV, *p* < 0.05, AD 0 µM vs. AD 50 µM RV, *p* < 0.05; *GSTZ1*: HC 0 µM vs. HC 50 µM RV, *p* < 0.001, AD 0 µM vs. AD 50 µM RV, *p* < 0.0001; *SIRT1*: HC 0 µM vs. HC 50 µM RV, *p* < 0.0001, AD 0 µM vs. AD 50 µM RV, *p* < 0.0001; *SIRT3*: HC 0 µM vs. HC 50 µM RV, *p* < 0.001, AD 0 µM vs. AD 50 µM RV, *p* < 0.0001; *SOD2*: HC 0 µM vs. HC 50 µM RV, *p* < 0.01, AD 0 µM vs. AD 50 µM RV, *p* < 0.0001; *TXNIP*: HC 0 µM vs. HC 50 µM RV, *p* < 0.001, AD 0 µM vs. AD 50 µM RV, *p* < 0.001; *VPS13C*: HC 0 µM vs. HC 50 µM RV, *p* < 0.1, AD 0 µM vs. AD 50 µM RV, *p* < 0.05) (Figure 5). In the case of *NFEL2L* and *CAT*, this upregulation was only present in the HC and in AD cell lines, respectively (*CAT*: AD 0 µM vs. AD 50 µM RV, *p* < 0.05; *NFEL2L*: HC 0 µM vs. HC 50 µM RV, *p* < 0.1) (Figure 5b,g).

AD lymphoblasts exhibited general upregulation of *CASP1* and *SOD2* and a downregulation of *CCS* compared to HC, according to two-way ANOVA (Table 3, Figure 5a,c,h).

These results generally confirmed the upregulation of antioxidant and anti-aging genes by RV in both HC and AD LCLs. However, the increased expression of *CASP1* in AD was not modified.

## 4. Discussion

In this study, we used LCLs derived from lymphocytes of AD patients and age-matched HCs to investigate the potential protective effects of RV and both Se (IV) and Se (VI) on ROS levels, generated at basal conditions and after an oxidative insult. We also studied the potential mechanisms of action involving modulation of antioxidant and anti-aging genes.

AD LCLs showed lower antioxidant defenses than HC LCLs, with higher ROS levels in response to different concentrations of oxidizing agents (H_2_O_2_ and FeSO_4_). The increased oxidative stress of AD LCLs is consistent with observations in many studies using fresh lymphocytes from AD patients [4,68], suggesting that the immortalized AD lymphocytes are a valuable model to test protective strategies. Furthermore, cellular level oxidative stress might be associated with pathology severity in the donor. In a cell model of familial AD by expression of *PSEN1* mutations in fibroblasts, we have previously seen higher basal and H_2_O_2_-induced ROS in the more aggressive mutations than in those causing a milder AD phenotype [69]. Here we used LCLs obtained at a moderate stage of sporadic AD and they showed distinct characteristics of oxidative stress. We may speculate that they show intermediate levels of AD-associated oxidative stress and are a sensitive model to test responses to interventions.

Interestingly, in the absence of oxidative insults, RV decreased ROS levels in both AD and HC LCLs. Given that oxidative stress is recognized as a risk factor for developing AD, this finding supports the potential preventive and therapeutic effects of this component by reducing the ROS burden not only in AD patients but also in healthy and at-risk populations. Consistent with this, it has been reported that RV can prevent the deleterious effects triggered by oxidative stress in neuronal cells and brain tissue of experimental AD models [70,71]. Likewise, under oxidizing conditions RV triggered a global protective response towards ROS in both AD and HC LCLs. Although the specific mode of action of RV needs further characterization, its antioxidant properties may contribute to the potent neuroprotective effects reported in AD mouse models [14,15,16], and to the promising effects reported in clinical trials with AD patients [19,20,72].

The Se compounds we used confirmed the antioxidant properties of this element in LCLs, although both Se (IV) and Se (VI) only exerted antioxidant effects in AD cell lines under H_2_O_2_ or FeSO_4_ treatment. This suggests that, compared to RV, Se (IV) and Se (VI) need a stronger oxidizing insult and higher basal levels of ROS to exert beneficial effects at the particular concentrations studied and time of exposure. In fact, whereas Se levels are decreased in AD patients [25,26], a recent study has shown that Se status is not associated with cognitive performance in a healthy population [73]. Similarly, no preventive AD effects have been reported for Se supplementation in a long-term supplementation trial [74]. Future studies of Se therapeutic properties would clarify whether there is increased protection under greater oxidant/antioxidant imbalance.

Regarding our transcriptional experiments, Se (IV) and Se (VI) did not significantly regulate any of the genes studied. Interestingly, we observed a general upregulation of the following genes caused by RV: *CAT*, *CCS*, *GSTZ1*, *NFE2L2*, *SIRT1*, *SIRT3*, *SOD2*, and *TXNIP*. Treatment with the highest concentration of RV (50 µM) consistently upregulated *CAT*, *CCS*, *GSTZ1*, *NFE2L2*, *SIRT1*, *SIRT3*, *SOD2*, *TXNIP*, and *VPS13C* in both AD and HC LCLs (there was a global RV effect for all these genes). Notably, *NFE2L2* (also known as *NRF2*) is a transcription factor involved in the activation of genes with antioxidant response elements (AREs) such as *SOD1* and many members of the glutathione S-transferase family [75], to counteract endogenously or exogenously generated oxidative stress. It also upregulates the expression of the histone deacetylases *SIRT1* [76,77] and *SIRT3* [78,79], according to several studies. The upregulation of *NFE2L2* could, therefore, be responsible for the upregulation of *GSTZ1*, *CCS* (a chaperone involved in the activation of *SOD1*), and both *SIRT1* and *SIRT3*. Similarly, *SIRT1* can enhance the activity of the NFE2l2/ARE pathway according to some authors, thus establishing a positive feedback [77,80,81]. These findings are consistent with previous studies of brain ischemia, Parkinson’s disease, and other pathological conditions showing that treatment with RV activates the NFE2l2/ARE pathway [82,83]. Furthermore, RV has been proven to activate *SIRT1* through the metabolic sensor AMPK, as indicated above, which is consistent with findings in animal studies [15]. Similarly, the increase in *CAT* expression is consistent with some animal and human studies showing an increase in both activity and expression of this enzyme as a consequence of RV treatment [84] or *SIRT1* overexpression [85]. SIRT1 is known to deacetylate SOD1 and promote its activity by facilitating its association with copper chaperone for SOD1 (CCS); therefore, the upregulation of both genes as a consequence of RV treatment may further contribute to ROS elimination by increasing SOD1 activity [86]. Interestingly, the gene in the sirtuin family *SIRT3* is a known key enzyme for the functionality of mitochondria and its decrease is linked to neurodegeneration such as AD [87]. *SOD2* is another mitochondrial enzyme that was upregulated by RV. Consistently, a previous study reported that *SOD2* expression was induced by RV-dependent activation of the PI3K/Akt and GSK-3β/β-catenin signaling pathways [88]. RV upregulation of *SIRT3* and *SOD2* would improve mitochondrial metabolism and energy efficiency, leading to decreased mitochondrial oxidative stress. In some cases, either HC or AD LCLs showed greater responsiveness to the upregulation of specific genes. For instance, the increase of *NEF2L2* by RV was significant in HC LCLs, whereas in the case of *CAT* the upregulation was significant for AD LCLs. This suggests that some genes could have a different response to RV treatment according to the cellular environment. For example, it is possible that *CAT* expression is sensitive to RV in a context of higher oxidative stress, and that *NEF2L2* constitutes a target of RV when used as a preventive approach or early treatment (i.e., when there is a low to moderate burden of ROS). Meanwhile, *TXNIP* is considered an intracellular amplifier of oxidative stress, since it is a negative regulator of the thioredoxin system; a major cellular thiol-reducing and antioxidant system. According to several authors, RV inhibits *TXNIP* expression [89]. Unexpectedly, we observed slight upregulation of this gene by RV, which is inconsistent with previous findings and could be counterproductive in the context of AD. Special attention should be paid to this issue in future studies. Finally, genetic studies have implicated loss-of-function mutations in the human *VPS13* gene in neurodegenerative disorders by causing defects in membrane lipid homeostasis [67,90]. The increase in this gene by RV might help prevent aging-associated lipid imbalances [91].

Regarding basal transcriptional differences between HC and AD LCLs, we observed that AD lymphoblasts exhibited general upregulation of *CASP1* and *SOD2* and downregulation of *CCS*. Consistently, CASP1 is activated in AD brains and overexpressed in monocytes from AD patients, and it is in fact considered a therapeutic target against age-dependent cognitive deficits and AD [92,93]. Elevated antioxidant enzyme levels have been reported before in AD patients [94], and the upregulation of *SOD2* might indicate compensatory upregulation of mitochondrial antioxidant defenses in response to oxidative stress in AD LCLs. Interestingly, a downregulation of CCS has been reported in several AD models, which diminishes *SOD1* activity and increases the expression of the enzyme β-secretase 1 involved in the amyloidogenic processing of the amyloid precursor protein (APP) [94,95,96,97]. We found that RV treatment upregulated *CCS* expression levels, thus reaching and surpassing those of HC LCLs under basal conditions.

Overall, this study confirms a derangement of oxidative defenses in lymphocytes from AD patients, indicating lower resilience to oxidative injuries and age-related oxidative stress, and supports RV as a more powerful compound, with more consistent and robust effects regarding antioxidant and transcriptional outcomes, than Se (IV) or Se (VI) under our experimental conditions. In particular, modulation of gene expression of important anti-aging (*SIRT1*, *SIRT3*, *VPS13C*) and antioxidant (*CAT*, *CCS*, *GSTZ1*, *NFE2L2*, *SOD2*) genes by RV seems to be partly contributing to its mechanism of action. The fact that protective mechanisms of RV are activated in cells from both healthy and diseased AD donors is in agreement with the activation of protective mechanisms against aberrant proteostasis in both wild-type and AD transgenic mice after chronic treatment with RV [15]. In that last study, we found that RV mechanisms yielded a strong neuroprotection against memory loss and AD pathology in transgenic mice and cognitive enhancement in wild-type mice. Here, the activation of anti-aging and antioxidant genes in our peripheral cell models further confirms that RV may be a potent protective agent and an inducer of resilience against aging and AD. Thus, in accordance with our findings and those of previous reports, RV should be studied further and considered a valuable nutraceutical candidate for early therapies aiming to prevent or delay the onset and progression of AD clinical symptoms.

Moreover, our findings reinforce the value of LCLs as a feasible model for understanding the protective mechanisms of nutraceuticals with antioxidant properties (such as RV) against the cumulative burden of oxidative stress and other cell alterations in AD.

## Figures and Tables

**Table 1:** Two-way ANOVA analysis of relative expression of genes involved in oxidative stress in AD and HC lymphoblasts treated with selenite (Se (IV)), selenate (Se (VI)), and resveratrol (RV).

Gene Name	Association with Oxidative Stress	Ref	Gene Symbol	Interaction			Treatment Effect			Disease Effect		
				F	(Dfn, DFd)	*p-*Value	F	(Dfn, DFd)	*p*-Value	F	(Dfn, DFd)	*p*-Value
Catalase	Catalase is an enzyme that protects aerobic cells from oxidative stress by catalyzing the rapid decomposition of hydrogen peroxide.	[47]	*CAT*	0.368	(3, 12)	0.7775	59.07	(3, 12)	***<0.0001***	0.00215	(1, 12)	0.9638
Copper chaperone for SOD1	CCS is involved in physiological SOD1 activation (one of the three superoxide dismutases responsible for metabolizing free superoxide radicals in the body), and its primary function is thought to be the delivery of copper to the enzyme.	[48]	*CCS*	0.3915	(3, 11)	0.7616	3.52	(3, 11)	*0.0524*	7.828	(1, 11)	***0.0173***
Alpha-galactosidase	GLA is an enzyme that hydrolyses the terminal alpha-galactosyl moieties from glycolipids and glycoproteins. Insufficient activity of GLA leads to accumulation of ROS.	[49]	*GLA*	0.0085	(3, 11)	0.9988	0.6197	(3, 11)	0.6167	3.679	(1, 11)	*0.0814*
Glutathione peroxidase 1	Glutathione peroxidase (GPX) is a class of antioxidant enzymes that catalyze the reduction of hydrogen peroxide to water. GPX1 overexpression is associated with enhanced protection against oxidative stress. GPX4 is the only glutathione peroxidase that accepts phospholipid hydroperoxides in membranes as an oxidizing substrate, and under conditions of glutathione deprivation, protein-thiol groups as the reducing substrate.	[50]	*GPX1*	0.3812	(3, 12)	0.7684	1.062	(3, 12)	0.4014	4.048	(1, 12)	*0.0672*
Glutathione peroxidase 4		[51]	*GPX4*	0.99	(3, 12)	0.4304	2.928	(3, 12)	*0.0770*	2.928	(1, 12)	0.1128
Glutathione reductase	GSR is an enzyme involved in the glutathione-dependent antioxidant system by reducing oxidized glutathione.	[52]	*GSR*	0.0076	(3, 11)	0.9990	0.953	(3, 11)	0.4487	1.935	(1, 11)	0.1917
Glutathione S-transferase zeta 1	GSTZ1 catalyzes glutathione-dependent isomerization of maleylacetoacetate to fumarylacetoacetate, which is the second-to-last step in the vital phenylalanine and tyrosine degradation pathway. Deficiency of this enzyme causes oxidative stress and activation of antioxidant response pathways.	[53]	*GSTZ1*	0.7762	(3, 11)	0.5313	10.16	(3, 11)	***0.0017***	6.139	(1, 11)	***0.0307***
Nuclear factor (erythroid-derived 2)-like 2	NFE2L2 is a transcription factor involved in the intracellular antioxidant machinery. This enzyme transactivates genes with antioxidant response elements (AREs), and it coordinates the expression of cytoprotective genes to counteract endogenously or exogenously generated oxidative stress	[54]	*NFE2L2*	0.05632	(3, 12)	0.9816	15.56	(3, 12)	***0.0002***	0.2812	(1, 12)	0.6056
Peroxiredoxin 5	PRDX5 is a novel thioredoxin peroxidase which directly promotes the elimination of hydrogen peroxide and neutralization of other reactive oxygen species.	[55]	*PRDX5*	0.5956	(3, 11)	0.6309	1.719	(3, 11)	0.2206	4.015	(1, 11)	*0.0704*
Superoxide dismutase 2	This gene is a member of the iron/manganese superoxide dismutase family. It encodes an antioxidant mitochondrial protein that binds to the superoxide byproducts of oxidative phosphorylation and converts them to hydrogen peroxide and diatomic oxygen.	[56]	*SOD2*	0.09583	(3, 12)	0.9609	14.93	(3, 12)	***0.0002***	0.6395	(1, 12)	0.4394
Thioredoxin interacting protein	TXNIP is a negative regulator of TRX, which plays a major role in maintaining the redox status. It is upregulated with aging; its overexpression shortens lifespan due to elevated oxidative DNA damage, whereas its downregulation enhances oxidative stress resistance and extends lifespan.	[57]	*TXNIP*	0.3262	(3, 11)	0.8065	3.863	(3, 11)	***0.0413***	7.142	(1, 11)	***0.0217***

*p*-values < 0.05 were considered statistically significant. DF stands for degrees of freedom. Bold and italic values correspond to statistically significant *p*-values and *p*-values < 0.1, respectively.

**Table 2:** Two-way ANOVA analysis of relative expression of age-related genes in AD and HC lymphoblasts treated with selenite (Se (IV)), selenate (Se (VI)), and resveratrol (RV).

Gene Name	Association with Aging	Ref	Gene Symbol	Interaction			Treatment Effect			Disease Effect		
				F	(DFn, DFd)	*p*-Value	F	(DFn, DFd)	*p*-Value	F	(DFn, DFd)	*p-*Value
Caspase 1	CASP1 is an inflammatory/apoptotic caspase involved in age-related cognitive impairment.	[59]	*CASP1*	0.02562	(3, 11)	0.9941	0.04521	(3, 11)	0.9865	8.679	(1, 11)	***0.0133***
E1A binding protein p300	EP300 is a transcriptional coactivator that mediates many transcriptional events including DNA repair. It also acts as a histone acetyltransferase to regulate transcription through chromatin structural changes. EP300 activity is attenuated in ageing mice.	[60]	*EP300*	0.06183	(3, 11)	0.9789	0.5095	(3, 11)	0.6838	1.135	(1, 11)	0.3096
Forkhead box O1	FOXO proteins represent a subfamily of transcription factors that act as key regulators of longevity downstream of insulin and insulin-like growth factor signaling. They are involved in stress resistance, metabolism, cell cycle arrest, and apoptosis.	[61]	*FOXO1*	0.05955	(3, 11)	0.9800	0.1451	(3, 11)	0.9307	1.252	(1, 11)	0.2870
Sirtuin 1	Sirtuins are nicotinamide adenine dinucleotide (NAD)-dependent protein deacetylases involved in oxidative stress, metabolism, inflammation, and other aging-related cellular processes. Lifestyle factors, including physical activity and diet, can influence healthspan via modifying the level of sirtuins.	[62]	*SIRT1*	0.5235	(3, 11)	0.675	4.022	(3, 11)	***0.0371***	0.7906	(1, 11)	0.3930
Sirtuin 3			*SIRT3*	0.07819	(3, 11)	0.9705	7.481	(3, 11)	***0.0053***	1.058	(1, 11)	0.3258
Sirtuin 6			*SIRT6*	0.2715	(3, 11)	0.8447	0.5264	(3, 11)	0.6732	0.04267	(1, 11)	0.5270
Synaptosome associated protein 23	SNAP23 regulates synaptic vesicle trafficking and fusion, and it is increased with aging and in AD patients.	[63]	*SNAP23*	0.04866	(3, 11)	0.985	1.97	(3, 11)	0.1771	0.7959	(1, 11)	0.3914
Transcription factor B1, mitochondrial	TFB1M is a dimethyltransferase involved in mitochondrial transcription. It is thought that this protein plays a role on the loss of mitochondrial function encountered in numerous disease states and the aging process.	[64]	*TFB1M*	0.7357	(3, 11)	0.5523	0.3503	(3, 11)	0.7898	2.256	(1, 11)	0.1612
TERF1 interacting nuclear factor 2	TINF2 is a component of the shelterin complex (telosome) that is involved in the regulation of telomere length and protection.	[65]	*TINF2*	0.1374	(3, 11)	0.9356	0.31	(3, 11)	0.8178	1.166	(1, 11)	0.3033
Toll interacting protein	TOLLIP is an adaptor molecule within the toll-like receptor (TLR) signaling pathway. It is involved in autophagy and clearance of protein aggregates and it is decreased in AD models.	[66]	*TOLLIP*	22.59	(3, 11)	0.200	46.49	(3, 11)	0.0516	0.009	(1, 11)	0.962
Vacuolar protein sorting 13 homolog C	VPS13A is a lipid transport protein. Its dysfunction in the nervous system is described to shorten life span and trigger age-associated neurodegeneration in animal models. Mutations in the human VPS13 genes are responsible for neurodevelopmental and neurodegenerative disorders.	[67]	*VPS13C*	0.2332	(3, 11)	0.8713	1.327	(3, 11)	0.3153	9.003	(1, 11)	***0.0121***

*p*-values < 0.05 were considered statistically significant. DF stands for degrees of freedom. Bold and italic values correspond to statistically significant *p*-values and *p*-values < 0.1, respectively.

**Table 3:** Two-way ANOVA analysis of relative expression of candidate genes involved in aging and oxidative stress in AD and HC lymphoblasts treated with different concentrations of resveratrol (RV).

Gene Symbol	Interaction			Treatment Effect			Disease Effect		
	F	(DFn, DFd)	*P* Value	F	(DFn, DFd)	*p*-Value	F	(DFn, DFd)	*p*-Value
*CASP1*	0.01959	(3, 84)	0.9962	0.2699	(3, 84)	0.8469	73.8	(1, 84)	***<0.0001***
*CAT*	0.1504	(3, 84)	0.9292	8.508	(3, 84)	***<0.0001***	0.6411	(1, 84)	0.4256
*CCS*	0.2515	(3, 84)	0.8601	10.34	(3, 84)	***<0.0001***	7.605	(1, 84)	***0.0071***
*GSTZ1*	0.2779	(3, 84)	0.8412	26.42	(3, 84)	***<0.0001***	0.1917	(1, 84)	0.6626
*NFE2L2*	0.03848	(3, 84)	0.9898	7.453	(3, 84)	***<0.0001***	2.202	(1, 84)	0.1416
*SIRT1*	0.4004	(3, 84)	0.7531	27.86	(3, 84)	***<0.0001***	0.8486	(1, 84)	0.3596
*SIRT3*	0.06445	(3, 84)	0.9785	23.87	(3, 84)	***<0.0001***	0.2658	(1, 84)	0.6075
*SOD2*	0.2123	(3, 82)	0.8876	20	(3, 82)	***<0.0001***	0.4858	(1, 82)	***0.0132***
*TXNIP*	0.002772	(3, 84)	0.9998	21.07	(3, 84)	***<0.0001***	0.5962	(1, 84)	0.4422
*VPS13C*	0.452	(3, 84)	0.7165	8.398	(3, 84)	***<0.0001***	0.01005	(1, 84)	0.9204

*p*-values < 0.05 were considered statistically significant. DF stands for degrees of freedom. Bold and italic values correspond to statistically significant *p*-values and *p*-values < 0.1, respectively.

## Supplementary Materials

The following are available online at https://www.mdpi.com/2072-6643/11/8/1764/s1, Table S1: List of primers and probe sets used for real time RT-PCR analysis.
