# Supplementary material for: Role of Resveratrol and Selenium on Oxidative Stress and Expression of Antioxidant and Anti-Aging Genes in Immortalized Lymphocytes from Alzheimer’s Disease Patients
Source: Nutrients. 2019 Jul 31;11(8):1764. doi: 10.3390/nu11081764 (PMC6723840; doi:10.3390/nu11081764)
Supplement: Supplementary file 1 [file nutrients-11-01764-s001.pdf]

Table S1. List of primers and probe sets used for real time RT-PCR analysis

Gene Symbol	Reference (ABI)	Reference Sequence ID
B2M	Hs00984230_m1	NM_004048.2
CASP1	Hs00354836_m1	NM_033295.3
CAT	Hs00156308_m1	NM_001752.3
CCS	Hs00192851_m1	NM_005125.1
EP300	Hs00914212_m1	NM_001429.3
FOXO1	Hs00231106_m1	NM_002015.3
GLA	Hs00609238_m1	NM_000169.2
GPX1	Hs00829989_gH	NM_001329455.1
GPX4	Hs00989766_g1	NM_002085.4
GSR	Hs00167317_m1	NM_001195104.2
GSTZ1	Hs01041668_m1	NM_001312660.1
NFE2L2	Hs00975961_g1	NM_001313903.1
PGK1	Hs00943178_g1	NM_000291.3
PRDX5	Hs00738905_g1	NM_181651.2
SIRT1	Hs01009006_m1	NM_012238.4
SIRT3	Hs00953477_m1	NM_001017524.2
SIRT6	Hs00213036_m1	NM_001321062.1
SNAP23	Hs01047496_m1	NM_130798.2
SOD2	Hs00167309_m1	NM_001322817.1
TFB1M	Hs01084404_m1	NM_001350502.1
TINF2	Hs01554307_g1	NM_001099274.1
TOLLIP	Hs04980661_s1	NM_001318512.1
TXNIP	Hs01006897_g1	NM_006472.5
VPS13C	Hs00419559_m1	NM_017684.4
